# Access to and use of medicines in the Annapurna region of Western Nepal and possible impacting factors

**DOI:** 10.1186/s40545-019-0172-3

**Published:** 2019-06-04

**Authors:** Bhuvan K.C., Susan Heydon, Pauline Norris

**Affiliations:** 1 School of Pharmacy, Monash University Malaysia, Jalan Lagoon Selatan, 47500 Subang Jaya, Selangor Malaysia; 2 School of Pharmacy, University of Otago, Dunedin, New Zealand

**Keywords:** Access to medicines, Quality use of medicines, Health systems, Society, Nepal

## Abstract

Improving access to medicines is a major healthcare challenge for low-income countries because the problem traverses health systems, society and multiple stakeholders. The Annapurna region of Nepal provides a valuable case study to investigate the interplay between medicines, society and health systems and their effects on access to and use of medicines. Government health facilities and international aid organizations provide some healthcare in the region, communities participate actively in healthcare organization and delivery, there is an important tourism sector and a mostly rural society.

This study investigates access to and use of medicines through health facility and household-based studies using standardised tools and through a series of structured key informant interviews with various stakeholders in health.

Overall, access to essential medicines at public health facilities was good, but this was not benefitting households as much as it should. People were using the private sector for medicines because of their perception about the quality and limited numbers of government-supplied free medicines. They utilised money from remittances and tourism, and subsidised healthcare from non-government organizations (NGOs) to access healthcare and medicines. A pluralistic healthcare system existed in the villages. Inappropriate use of medicines was found in households and was linked to the inadequate health system, socioeconomic and sociocultural practices and beliefs. Nevertheless, the often disadvantaged Dalit users said that they did not face any discrimination in access to health services and medicines. The government as the main stakeholder of health was unable to meet people’s health services and medicines needs; however, health aid agencies and the local community supported these needs to some extent.

This study shows that the interconnectedness between medicines, society and health systems impacts the way people access and use medicines. Improving access to medicines requires an improvement in public’s perception about quality, actual coverage and appropriate use of medicines and health services via collaborative contributions of all stakeholders.

**Electronic supplementary material:**

The online version of this article (10.1186/s40545-019-0172-3) contains supplementary material, which is available to authorized users.

## Background

The Annapurna region is a cluster of rural hilly villages spread across five districts and 57 villages development committees of the Western region of Nepal [1]. It is located more than 200 km from Kathmandu and provides a suitable context (case) to investigate access to and use of medicines by exploring the interconnectedness between medicines, society, health system and its stakeholders. The region consists of rural villages with a population of more than 120,000 made up of eleven major and some minor ethnic groups [1]. Some villages of the Annapurna region have received international aid over many years for development and delivery of health services and medicines. These include health post construction and the Community Drug Program in Ghandruk, Indian aid for building a health post in Sikles, American Himalaya Foundation aid for a health clinic in Lomangthang, a missionary NGO run hospital in Lamjung, and health clinics in Manang and Mustang [1–6]. Likewise, local community involvement in the development and delivery of healthcare services and medicines has been an important feature in some of the villages [1]. The major referral public hospitals and private healthcare providers in the Annapurna region are located in the Pokhara Valley of the Western Development Region. Most people have to walk for several hours or days and use a locally available vehicle to reach these services. Another important feature of the Annapurna region is tourism, which provides better economic opportunities for people and has helped in infrastructure development in the region [7, 8]. Tourism has also served the health needs of local people through a high altitude medical post in Manang village [9].

A pluralistic healthcare environment exists in the Annapurna region as people use Ayurvedic medicines, herbal medicines, Tibetan medicines, faith healers and home based remedies. The literature show that the rural lifestyle, socio-cultural and socio-religious belief systems have affected the way people access and use medicines [10–12]. Likewise, a mixed demography and socioeconomic factors such as the remittances sent by migrant workers, female heads of households, contributions from the families of soldiers in the Indian Army and British Army to local health systems and health literacy too, have an impact on the way people use medicines in the region [4, 5]. These contextual factors contribute to financing of medicines, healthcare seeking, access to medicines, use of traditional medicines and medicine-taking [4, 5].

The literature also show that there is a linkage between medicines and local community efforts, medicines and international aid, medicines and an inadequate health system and other societal features in the Annapurna region [1–5, 13–15]. However, there is a dearth of studies investigating the interaction between medicines, society, health systems and stakeholders, and the impact on access to and use of medicines. Several important aspects of access to medicines are not known. The state of access to medicines such as physical availability, geographical accessibility, affordability and quality of essential medicines and health services are not known. Likewise, how people access these medicines via the formal and informal health system in the Annapurna region too is not known. Whether people use these medicines in the right manner has not been explored. Furthermore, whether local stakeholders of health contribute to access to medicines or not too has not been explored yet. Thus, in this article, we investigate access to and use of medicines through exploring the interplay between medicines, society, health systems and stakeholders. The objectives of this study are to assess the state of access to and use of medicines at health facility level and households level, explore the contribution of health system stakeholders towards improving access to medicines and understand how society, health system and its stakeholders interact to affect access to and use of medicines in the Annapurna region.

## Method

This study uses a case study approach. The whole of the Annapurna region was taken as a case and four specific villages out of 57 village development committees were selected as the subunits of the case. As shown in Table 1, these villages were selected so that the results could then be drawn together to yield an overall picture. The research setting involved four villages: Dhampus, Ghandruk and Manang (villages on or near tourist trails) and Rivan village (off the tourist trail). Each of these village had either a health post or sub health post. These villages were selected purposely to achieve the study objectives. These villages had active local community that have contributed towards local health development, received international aid for health, NGOs working in the area of health, contribution via tourism and other elements which allowed the investigation of access to and use of medicines taking into account of the interplay between medicines, society, health system and its stakeholders.

**Table 1:** Village profiles

Non-tourist village		Tourist village		
	Rivan	Dhampus	Ghandruk	Manang
Location	North of Kaski (900 m–2000 m and above)	North of Kaski (800 m–1600 m and above)	North of Kaski (1000 m–3000 m and above)	North part of Manang (3000 m and above)
Population (Households)	364	620	1102	131
Average household size	3.66	4.09	3.87	4.81
Total population	1332	2537	4265	630
Source of income	Agriculture and remittances	Agriculture, remittances and tourism	Agriculture, remittances and tourism	Agriculture and tourism
Access to healthcare services	Sub-health post, > 2 h to reach hospital	Sub-health post, < 1 h to reach hospital	Health post with pharmacy service, > 4 h to reach hospital	Health Post, NGO clinic, Tourist medical post, > 2 days to reach hospital

### Sub health post

Smallest primary healthcare unit managed by a district health office. It provides basic health service and limited (around 25) free essential medicines, run vaccination and reproductive health programs and other preventive healthcare programs. A sub health post is headed by an auxiliary health worker and two other staff: a village health worker and office helper.

### Health post

Health posts are a level above sub health post and provides basic healthcare and around 30 free essential medicines, run vaccination and reproductive healthcare programs and other preventive health programs. A health post is run by a senior auxiliary health worker, an auxiliary midwife nurse, village health worker and an office helper.

The sub health post and health post remains open from 10:00 am to 3:00 pm and are managed by local health facility managed committees while resources for health care and staff salaries are paid by the government via the respective district health offices. All local people can access the health post and sub health post and all services and medicines are provided free of the cost.

To explore access to and use of medicines and its linkage with health system and society health facility based surveys, households based semi-structured interviews, and key informant interviews with stakeholders of health were carried out in each of these four villages.

1. Health facility based study on access to medicines:

The access to medicines health facility based study form was adapted from the WHO operational guide for level II study (Additional file 1, The WHO level II study uses systematic health facility and household based surveys to measure access to and rational use of medicines [16]. The WHO level II indicators mainly measure a) access, by examining the availability and affordability of essential medicines (in the public sector and for the poor), b) quality, by examining the presence of expired medicines on pharmacy shelves, how medicines are handled and how well are they conserved in health facilities, and c) rational use, by examining prescribing and dispensing pattern and whether strategies such as the Standard Treatment Guidelines (STGs) and Essential Medicines Lists (EMLs)] [16] are implemented or not.] [16]. The health facility based access to medicines study tool was finalised following a pilot study and included three parts (Additional file 1: Appendix I: a) interview with health post incharge (person in charge of the health post) on state of access to medicines; b) health facility survey covering various dimension of access and c) exit interviews with health service users and collection of information on medicines prescribed and dispensed.

The health facility based study on access to medicines was carried out in two phases, phase I in January–April 2014 and phase II in November–December 2014 to capture the medicines access situation in different fiscal quarters of the Nepal government. The study was conducted in Dhampus Sub-Health Post, Rivan Sub-Health Post, Ghandruk Community Health Post and Tangki-Manang Health Post. Data collection was carried out by the researcher and a research assistant.

2. Semi-structured interviews on households’ medicines use:

Following the health facility based study on access to medicines semi-structured interviews was carried with households in the respective four villages.

A semi-structured interview schedule was developed based on a literature review of social issues in medicines use in Nepal and adapting the WHO operational guide for level II study (Additional file 1: Appendix II). It was finalised following a pilot study in a village similar to the research site. The semi-structured interview schedule covered: sociodemographic profile of households, how they access medicines from different sources, what problem do they face in accessing medicines for both general use and chronic health problems, how they use medicines in households, usage of traditional medicine, and state of Dalit’s access to medicines.

### Sampling of the study population

The Nepal Population and Housing Census 2011 was used to obtain information on the population and the total number of households in Dhampus, Rivan, Ghandruk and Manang Village Development Committee (VDCs). Based on the response during the household interviews and the type/range of answers for interview questions in the pilot study, data saturation was achieved after six to nine interviews. Thus, a sample target to cover more than 5% of the households from a village by including households from all the nine wards of a village was set. However, the final number of households interviewed was adjusted based on the actual interview responses and the location of Dalit households in that village.

Ward is the smallest unit of each village. From each ward households were selected randomly from different households’ clusters. However, if any ward contain Dalit households cluster then households were selected from those Dalit households cluster to include Dalit households. At least six Dalit households were selected and interviewed from each village as data saturation was achieved around six households. A few more Dalit households were included if any village contain bigger Dalit population.

Semi-structured interview with households were carried by the researcher and research assistant in Nepal language. The interviews with households were carried out in their respective houses. Interview transcript were hand written in Nepali language and later on translated into English. The response to each question was read back to the household member to confirm their response. Each households interview lasted for 30 min.

Data collection period: The data collection was carried out from January–April 2014. A total of 134 households (134 houses) (Dhampus - 31 households, Rivan - 30 households, Ghandruk - 55 households and Manang - 18 households) were interviewed in the study.

3. Key informant interviews with various stakeholders in public health:

To know different stakeholders’ perspective on access to medicines, key informants were identified and interviewed from the study region. Key informants identified were members of village development committee, members of health facility management committee, members of female community health volunteer, government health workers and any other community members and staff of NGOs/INGOs involved in public health project.

A key informant interviews schedule was developed following an extensive document analysis and literature review, and a reconnaissance visit of the study site. We looked at documents from the WHO, Ministry of Health Nepal and reports from different NGOs/INGOs working in the area of healthcare delivery in Nepal.

The interviews were qualitative and in-depth, each interview lasting about one hour. The interviews were carried out in a flexible interview process, where topics were dealt with as they came up rather than following a fixed order, and so allowed the interviewees to express his/her view in a flexible and coherent manner. Different interview guides (see Additional file 1: Appendix III) were used depending on who the informant represented (community, government and NGO/INGO/humanitarian aid agency/faith based Organization).

### Data collection

Data collection was carried out in two phases: phase I in January–April 2014 and phase II in November–December 2014. The key informants interview was carried out in their office. Fifteen key informants were interviewed. Some of the interviews were audio recorded while some were hand written as some of the interviewees decline the interviews to be audio recorded.

### Data analysis

The Nepali language transcript of the interviews from health facility, households and key informants were translated into English by the researcher who is a native Nepali speaker fluent in both English and Nepali. In addition, the Nepali to English language translation was reviewed and verified by professional translator during the process of writing the PhD monograph and the same information has been used in this manuscript.

The health facility based study contained both quantitative and qualitative data. Both the quantitative and short qualitative data were entered into separate sheets in Excel®. These data were entered into xls file as it would allow for those information to be categorized under different heading and subheading and analysed later on. The quantitative information about access to medicines (from both health facility observation and health service users interview) was analysed using simple descriptive statistics such as frequency and average. This was presented as a percentage to reflect various indicators of access viz. availability, accessibility (geographical), affordability (loss of pay due to illness) and acceptability, appropriate provisions of medicines storage and handling, and rational use of drugs (use of medicines in accordance with the patient’s clinical need, and in the right dose, frequency and duration, and at a cost that is affordable to the patient).

The qualitative information from both the health facility based study and semi-structured household interviews was analysed using a basic thematic analysis. The final result was presented as categories/themes that reflected the interview.

The transcript from the key informant interviews was imported into the software QSR NVivo 10 for content coding and analysis. The interviews were analysed as per the themes and the final result was presented as categories that reflected the key informants’ interviews.

## Results

The section first covers access to medicines in relation to the Nepalese health system. It then gives an account of the overall state of essential medicines in the Annapurna region and lastly the bigger picture regarding medicines, society, the Nepalese health system and their interconnectedness.

### Public health system in the Annapurna region and access to medicines

#### Households’ characteristics and medicines use

The majority (58%) of the households in the studied villages of the Annapurna region were headed by females. Most (72%) of the household heads had either not finished school level education or not undertaken any formal education. The main sources of household income in three villages (Rivan, Dhampus and Ghandruk) were a combination of remittances and agriculture (40%). However, in Manang village the biggest source of income was the combination of business and agriculture (44%).

The overall state of access to basic medicines in the studied villages Annapurna region was good as indicated by high availability, no expired medicines, few stock-out days, free medicines and better geographical accessibility for the majority of health service users (Table 2). However, the majority of health service users expressed dissatisfaction with the quality of medicines and health services, and the limited number of medicines, and it was consistent throughout all the studied villages. Nevertheless, there were some variability in access to medicines across the villages. As shown in Table 3 below, Ghandruk health post had 3.13 days of stock-out of medicine while Manang health post had no proper log book of medicines availability over last six months. Likewise, health service users at Rivan village had highest loss of pay of USD 7.35.

**Table 2:** Access to and rational use of medicines

Parameters	Results	
Physical availability (*N* = 19 tracer medicines)	93%	
Presence of expired medicines	0%	
Stock-out of medicines (in last 6 months to 1 year period)	1.04 days	
People living at a distance of less than one hour from the health facility	67%	
Waiting time for health services users in health facility		
Less than 30 min	100%	
More than 30 min	0%	
Affordability (basic medicines were available for free)		
Number of days of work missed due to illness	2 days	
Loss of pay due to illness	USD 5.42	
Acceptability		
Satisfaction with the recent health post visit	Yes	No
	54%	46%
Satisfaction with the medicines	21%	79%
Average number of medicines per prescription	1.55	
Percentage of medicines adequately labelled	9%	
Percentage of prescription with antibiotics	39%	
Percentage of health service users who knew how to use medicines dispensed to them	96%

**Table 3:** Access to medicines situation in individual villages

Dhampus SHP	Ghandruk HP	Manang HP	Rivan SHP
Physical availability (N = 19 tracer medicines)			
100%	89%	82%	100%
Presence of expired medicines			
0%	0%	0%	0%
Stock-out of medicines (in last 6 months to 1 year period)			
0 days	3.13 days	NA	0 days
People living at a distance of less than one hour from the health facility			
59%	53%	100%	56%
Waiting time for health services users in health facility			
Less than 30 min			
100%	100%	100%	100%
Affordability (basic medicines were available for free)			
Number of days of work missed due to illness			
1.23 days	1.42 days	1.5 days	2 days
Loss of pay due to illness			
USD 4.39	USD 4.59	USD 5.35	USD7.35
Acceptability			
Satisfaction with the recent health post visit			
Yes =53%	Yes =95%	Yes =0%	Yes =69%
No =47%	No =5%	No =100%	No =31%
Satisfaction with the quality of medicines			
Yes =29%	Yes =37%	Yes =0%	Yes =20%
No =71%	No =63%	No =100%	No =80%
Average number of medicines per prescription			
1.58	1.47	1.5	1.68
Percentage of medicines adequately labelled			
24%	0%	0%	13%
Percentage of prescription with antibiotics			
41%	32%	50%	32%
Percentage of health service users who knew how to use medicines dispensed to them			
94%	95%	100%	94%

Dalit health service users said they did not face any difficulties in accessing health services and medicines. Furthermore, the Dalit households’ members in all of the studied villages said that they do not have problem in accessing health services and medicines from health facilities because of their sociocultural background. They said that the healthcare providers’ treat them good. However, the Dalits had problem with quality and coverage of health services and medicines supplied by the government health facilities and the need to buy medicines from private drug retailer. Nevertheless, health services users and households from non-Dalit group also had these problems regarding quality and coverage of health services and medicines.

We do not have any problem in accessing health service and medicines from health post … But, sometime we do not get medicines we want at the health post … [so] we have to buy medicines from the private drug retailer in the next village. (Dalit household member Rivan village #5)

As shown in Table 2, there were some problems with the rational use of medicines, such as 39% of the prescription containing antibiotics, only 9% of the medicines being adequately labelled (a medicine is considered adequately labelled if it contained complete information about the patient, the dosage, frequency and duration of medicine, instruction on how to take the medicine and how to store it) none of the health facilities having STGs and EMLs (Table 4). Again, there were variation within villages. As shown in Table 4, antibiotics per prescription was higher in health posts of Dhampus and Manang village, and labelling of medicines was relatively better at Dhampus sub health post. However, none of the health services users were prescribed injections and 96% of the users knew how to use the medicines dispensed to them. Also, only 62% of criteria for adequate handling and conservation condition of medicines was met by the dispensing room with 59% of the criteria being met by the storage room. It was worst in the case of Rivan Sub-Health Post where only 12.5% of the criteria were met by the storage room.

**Table 4:** Stakeholders in health and access to medicines

Stakeholders	Contributions	Problems and Issues
Local community	Provided administrative and managerial support via the health facility management committee	No such support in Rivan village due to lack of resources with the village development committee
	Supported health post on infrastructure development, equipment and programs	
	Supported delivery of medicines and health services via the female community health volunteer	
NGOs and INGOs	Supported public health project and pharmacy services	Lack of coordination among NGOs and INGOs and with the health system
	Directly provided medicines and health service via missionary hospitals	Decline in international funding and sustainability issue
	Provided much needed secondary care, long term preventive health and rehabilitation support	logistics and bureaucratic problems in implementing projects
Tourism	Direct support for essential medicines and health services via donations and health camps	Medicines and health use by tourists affect local people’s medicine taking behaviour
	Infrastructure support	
	Indirect support by providing income via tourism business	
Government Health System	Provided limited free medicines and basic healthcare	
	Provided vaccination, reproductive health services and preventive healthcare programs	Problem with quality and limited number of free essential medicines.

### Quality of product and services

The quality and adequacy of health services and medicines were perceived to be the main problems and the central concern in all villages. During both health facilities based interviews and household based interviews health service users expressed dissatisfaction with the quality and the limited number of free medicines available from government health facilities. People said that the free medicines are not of good quality, not powerful enough to treat their illness and that the free medicines available from government health facilities did not work for them. Some even said that they were like “mud” i.e. with no effect.

We are happy that government has provided essential medicines for free but the medicines are not of good quality … only a limited number of medicines are provided by health post. So I think that government should provide good quality medicines … [and] increase the number of medicines. (Dalit household member Dhampus #5)

Health service users and households also said that the free medicines provided by the government are too few and do not meet their medicines needs. They said that they have to buy medicines from the private sector, paying a high price for medicines that compromised their monthly budget for essential goods.

We do not have any problem in accessing health service and medicines from health post … But, sometime we do not get medicines we want at the health post … [so] we have to buy medicines from the private drug retailer in the next village. (Health service users Rivan village #11)

Most households and health service users in Dhampus, Rivan and Manang said that they preferred and used private health facilities for medicines and health services. However, in Ghandruk village health service users said that they preferred and used the government health post. Ghandruk health post was running a community managed pharmacy in the health post where people could buy approximately 100 different essential medicines (compared to the 25 available in the other villages).

Inappropriate handling and use of medicines was the problem with majority of households of the studied village of Annapurna region. For example, people cut their treatment short as they could not buy medicines for the full duration of treatment, they took allopathic medicines together with medicines from other medical traditions, they believed antibiotics were powerful medicines and they also believed that taking more medicines means more side effects. Likewise, non-adherence with medicines instructions was also found in majority of the households. This non-adherence was found especially with antibiotics, medicines requiring multiple-daily dosing and medicines that were given for a longer duration. Consuming medicines without checking the expiry date was also found in the households.

The reasons behind non-adherence were work burden, forgetfulness, inconvenience of multiple-daily dosing, side effects of certain medicines and prior experiences of not complying with medicines’ schedules.

Oh! Medicines taking is such a problem for me that [it’s such a big problem for me] I cannot say … When medicines are given for three or more times a day for longer period of time like 1 week … I follow the exact schedule for the first few days … then, as my health condition improves … I start working in field and I forget to take some dose of medicines … I usually forget to take mid-day dose of medicines. Sometime when I have to work in field I skip the day time dose of the medicines. (Household member Rivan village #3)

Medicines, both currently used in the households and those kept for future use, were inappropriately labelled and stored within children’s reach. These included antibiotics.

Households in the Annapurna region were using medicines from various systems of medicine. More than half of the households said that they use various types of medicines such as Ayurvedic medicines, herbal medicines, faith healers, and Tibetan (Amchi) medicine, along with allopathic medicines. Some 12% of households were using a variety of these medicines during the time of the study. Anti-gastric powder (for hyperacidity), Ayurvedic massage oil (for joint pain), hair oil (for hair loss), Chiraito syrup (for diabetes), Bojho rhizome (for sore throat) and Sancho (for body ache, common cold) were some of those commonly used. In some 50% of the medicines the dose, duration and method of administration were not known and 43% were used together with some form of allopathic medicines.

### Medicines used for chronic health problems in households

Forty-two percent of the households in the studied village of Annapurna region had members with chronic health problems such as diabetes, hypertension, gastrointestinal problems (gastritis and hyperacidity) neuromuscular problem, and asthma. Eighty-two percent of the households with members who had chronic health problems were taking medicines while the remainder were not as they could not afford the treatment cost. Eighty-five percent of the households relied on out-of-pocket financing to buy medicines for these chronic health problems. Some 15% of households used alternative sources for buying medicines for chronic health problems such as free medicines from aid funded NGO clinics, veteran healthcare beneficiary services for the Indian and the British Army veterans and their dependents, and free medicines distributed from the government.

### Sources of medicines and financing

Households in the studied villages of the Annapurna region obtained/bought various medicines such as Ayurvedic medicines, Tibetan medicines and herbal medicines from several outlets in the Pokhara Valley. They accessed faith healers and home-based remedies through social networks and in an informal setting at the community and household level.

Regarding allopathic medicines, people in Dhampus and Rivan villages were able to get some twenty-five essential medicines (none for chronic diseases) from the local sub-health post and people in Ghandruk and Manang were able to get thirty essential medicines from local health post. Alternative resources such as a community-managed pharmacy was available in Ghandruk village, and an NGO clinic and tourist health facility were available in Manang where people could get a wide range of medicines. Households in all the four villages obtained the rest of the medicines they needed, including medicines for chronic health problems, from private drug shops and hospital pharmacies which were located in market towns at a distance of more than one hour or in Pokhara city located at a distance of more than two hours. People have to both walk and pay to use a locally available vehicle to reach these places. Households in the Annapurna region, therefore, financed health services and medicines through various methods such as the government free health services and medicines, free and subsidised health services and medicines provided by health aid agencies such as NGO clinics, missionary hospitals, and frequently through out-of-pocket payment.

### Stakeholders and the relationships between medicines and society

The public health system, local community, NGOs and tourism were four significant stakeholders involved in access to healthcare and medicines. The local community, NGOs and tourism sector interacted with society and health system at different levels in the process.

The local community financially supported health post construction and equipment for Dhampus sub-health post. Similarly, the local community together with a local NGO helped Ghandruk community health post to run a pharmacy, set up a laboratory and hire extra healthcare workers for public health projects. The local communities in all four villages provided administrative and managerial support to their health post through management committees. The Female Community Health Volunteers (FCHVs), who come from the local community, were directly involved in the delivery of reproductive health services, mass drug administration of albendazole and diethylcarbamazine, routine immunisation and Vitamin A distribution for children, basic medicines through village health clinics and other public health projects.

… It all goes to the continued effort of FCHVs to educate people about the importance of health issues that people now are quite supportive towards public health activities. They support and participate actively in programmes like Vitamin A campaign, immunisation campaign, albendazole medication campaign etc. But I agree that there is still some work [more work] to do towards improving utilisation of family planning services. (FCHV Ghandruk #1)

However, there was no significant contribution from the local community in Rivan village in terms of donations and other support for the health post. Furthermore, the local community in Rivan did not have any economic resources such as local funds, donations from local people and tourists, and international funding that were available in tourist villages.

An NGO funded by Australian donors supported a public health project and pharmacy services in Ghandruk village. International aid-funded NGO clinics also provided free health services and medicines in Manang village. However, key informants also talked about issues with health aid agencies such as lack of coordination with the government, a decline in international funding, sustainability concerns, and logistics and bureaucratic problems in delivering health services in the villages of the Annapurna region.

So far as finance part is concerned we are finding it hard to keep of the cost … The hospital and leprosy program is always struggling for funding. The increasing cost and patient influx … has increased our cost and our financial reports suggests that we have to generate some income locally to survive the hospital and manage the overall expenses … We are also collaborating with international donors and trying to get some funds but it will be a slow process.

(Staff from an INGO #1)

Tourism provided much-needed healthcare and medicines for local people in Manang through a high altitude medical aid post. Tourists also donated medicines and equipment to some of the health facilities of the Annapurna region and carried out health camps occasionally in some villages.

Tourism business provided income opportunities for households in Ghandruk, Rivan, Manang and several other villages of the Annapurna region and enabled people to access healthcare and other services in the private sector. However, health camps and medicines used and donated by tourists also affected the way local people used healthcare and medicines. Households visited their local health facility demanding a certain type of medicine such as analgesic gels, antibiotic powder, medicated patches and many other dosage forms which they got from tourists during health camps.

The government’s free basic healthcare programme provided limited free medicines and health services to the villages of the Annapurna region via a health post, sub-health post and primary healthcare centre. Moreover, the government also had programmes such as village health clinics and a telemedicine service. However, the key informants from the local community said that there were serious problems with the quality and coverage of public health services and medicines in their village, in the Annapurna region. They said that the government should improve the quality and coverage of health services and medicines, and should upgrade government health facilities so that people can get good primary healthcare services at the village level.

## Discussion

The overall state of access to medicines in the studied villages of the Annapurna region was good in comparison to a Nepalese study (2014) in Kathmandu, Banke and Bardia districts which reported 50–77% availability and 30% stock out of medicines, and international studies in Kenya (2009) with 46 days stock-out of medicines and in Jamaica (2010) with 90 days stock-out of medicines and unaffordable medicines prices [17–19]. However, availability and stock out of medicines in the studied villages of Annapurna region was comparable to other places of Nepal when compared to a study from Adhikari et al. carried out in 2014 which reported 92.44% availability and 0.324 days of stock-out of medicines [15]. Study from Adhikari et al. included both primary healthcare facilities and private drug retailers and was carried out in all three geographical region of Nepal the mountains, hills and plains while our study was carried out in the hills and mountain region only and did not included primary health care facilities and private drug retailers due to their absence in the studied villages [15]. This improvement could be due to both better logistics provision (better physical infrastructure of health facilities, good walking trails, access to porters and mules to carry goods and additional financial support from VDCs) in the Annapurna region because of sustainable tourism and local development initiatives, and improvement in the delivery of free basic healthcare services over the years since their inception in 2007 [8, 17]. Furthermore, the finding that Dalits did not appear to face discrimination in accessing health services and medicines shows signs of improving access. Whether this is the case throughout the region is not known, but the improvement in Dalits’ access may be due to better socioeconomic conditions of the villagers (because of tourism business, remittances and access to education), targeted government programmes for Dalits and the better demographic profile of Dalits in villages as non-Dalits continue to migrate to urban areas [2, 19].

Appropriate use of medicines in the public health facilities of the studied villages of Annapurna region was better with no injection use at health post and sub-health post level and a lower level of antibiotics prescribing compared to previous studies from Nepal [14, 20]. This may be due to things like households’ preference and use of private health facilities for serious illness which might require antibiotics and injections, the limited number of medicines available at government health facilities (including very few antibiotics and injections), but also importantly improving awareness among Primary Health Care (PHC) workers regarding antibiotics and injection use due to the Ministry of Health’s campaigns. However, inadequate labelling and dispensing of medicines, deficient storage and handling of medicines, and the lack of Standard Treatment Guidelines and an Essential Medicines List shows that further improvement in medicines use standards are needed. The inappropriate handling and use of medicines noted in this study were similar to the findings of the studies carried out in India, Kenya and Jamaica [18, 19, 21]. This may be because of the similar demographic and socioeconomic characteristics of the households in these countries such as rural locations, the majority of household heads lacking formal education or not completing school level education, poverty and poor social infrastructure [18, 19, 21].

The majority of the health service users were unsatisfied with the quality of medicines and health services, and the limited number of medicines and it was worst in the case of Manang village which was located in the most remote place and Rivan village which is a non-tourist village lacking access to alternative resources. A study by Patel et al. also showed that South African consumers considered that both generic medicines and state supplied free essential medicines were of poor quality and treated them with suspicion [22]. The expressed concern in the Annapurna region about the poor quality of medicines from public health facilities might have several complex reasons such as the substandard medicines as evident from previous cases in Nepal of substandard iron capsules and misoprostol tablets being distributed, people’s perception about free medicines that they are not of good quality, poor logistics and infrastructure hampering medicines’ quality during storage and distribution [23, 24]. A study by Ferrario et al., highlights the role of regulatory affairs affecting the availability of quality medicines in Moldova and it might have a role in Nepal case as better pharmaceutical regulatory ecosystem favours the manufacturing and procurement of quality essential medicines [25]. Further lab based analytical studies on the quality of free medicines are required to know more about the quality of free essential medicines in Nepal. Though the availability of basic medicines was good, the total number of medicines available at the health post and sub-health post was less because these facilities were provided with only 18 and 25% of the essential medicines list (*N* = 139 medicines essential medicines for primary healthcare level) that were meant to be used at primary healthcare level. The total number of medicines available at the primary healthcare level in Nepal was much smaller than the range of essential medicines available at PHC level in Sri Lanka [26, 27]. There is, therefore, a need to improve both the quality and coverage of medicines and improve service delivery in public health facilities.

Inappropriate handling and use of medicines and non-adherence with medicines instructions were major issues for villagers in the studied villages of Annapurna region. These problems were rooted in socio-economic factors, socio-cultural practices and geography. A study by Heydon with Sherpa of the Mt. Everest region of Nepal also shows that cultural practices related to health prevention and people’s perception regarding efficacy and appropriateness of medicines are important factors when taking medicines [12]. Studies from countries such as Ghana, Jamaica, Uganda and India have also reported inappropriate handling and use of medicines by households [19, 28–30]. However, in Ghana’s case non-adherence was not related to affordability but was related to inappropriate treatment practices among prescribers while in Uganda’s case it was related to unaffordable treatment and low availability of medicines [28, 29].

Evidence from the Annapurna region showed that elements like tourism have sociocultural effect on healthcare seeking practice and medicine taking behaviour of local people. The health aid and health camps provided by tourists were helping the local people with medicines and healthcare. However, it was also affecting the local people’s perception and practice regarding healthcare and medicines. For example, the local people demanded certain type of medicines such as ointments, creams, capsules and antibiotics from the local health facilities as they received these medicines from the tourists and they found these medicines to be quite effective. A study by Susan Heydon in the Everest region also suggest that tourism has affect the spread of modern medicine and healthcare seeking practice of local people [12].

A significant portion (42%) of households had members with chronic health problems. Most used out-of-pocket payment for accessing healthcare and buying essential medicines, including medicines for chronic health problems. Households used alternative resources such as NGO clinics, a tourist health facility, missionary hospitals and veteran healthcare beneficiary schemes of the Indian and the British Army to access healthcare and medicines. Some of these resources are quite unique to the Annapurna region. Since the public health system had real and perceived challenges with quality and coverage of health services and medicines, the poor and vulnerable groups had difficulties accessing treatment for both chronic and other healthcare problems, despite the fact that the current free basic healthcare programme targets these groups.

There is a need for the government to improve the free basic healthcare programme by improving the quality and coverage of health services and medicines and this study suggests that in order to do this the government should liaise with local community members in the delivery process. The local communities have contributed significantly to the development and delivery of health services and medicines in Nepal. Studies from India, Kenya, Mali and Cambodia also show that local communities can contribute to the delivery of health services (reproductive health services, pneumonia, tuberculosis etc.) and help to improve it [30–34]. However, the extent of contribution from local community need to be carefully planned and executed without overburdening them. Likewise, health aid agencies also contributed to the development and delivery of much-needed health services to the rural villages of the Annapurna region. However, effective implementation and coordination, and the overall functioning of aid funded healthcare programmes has often been contested locally, nationally and internationally [5, 7, 35–37]. It is important for Nepalese health aid agencies to improve coordination with other agencies and bureaus, to minimise duplication of resources and to improve the implementation of their healthcare programmes. Tourism also contributed to access to health services and medicines both directly and indirectly; but, it was limited to certain tourist villages only. By and large, the government needs to further improve the quality and coverage of health service delivery with a special focus on rural villages with a poor socio-economic profile.

### Strength and limitation

The main strength of this study is the exploration of health system, local community and other stakeholders’ role in access to medicines in detail at the village level. This study pulls information from health facilities, local households, local community members, international agencies and local NGOs, government’s health sector and tourism so as to know the state of access to and use of medicines and how these elements are interconnected and how they contribute towards access to healthcare and medicines. Thus, it brings a comprehensive view on the issue of access to medicines and how different stakeholders in health contribute towards access to healthcare and medicines at a local level in a Nepalese village.

This study was carried out as a part of PhD project and was limited to few villages of the Annapurna region. This study included only five villages (actually four villages but Manang health post was set up for both Tangki and Manang villages so four health facilities representing five villages were studied) of the Annapurna region and the health facilities located there. Inclusion of more villages and more health facilities could have improved the scope of the study. Furthermore, none of the studied village in this study had private drug retailers. Information about the availability of private drug retailers could also have added to the quality of the study. Furthermore, the households included in this study were determined based on data saturation and resources available for the study. Inclusion of more households as a representative of its total population could have given a fairer representation of the minority population such as Dalits and it could have added to the richness of this study.

## Conclusions

This study shows that access to basic medicines in the Annapurna region is good. Both an improving public health system and better logistics in this tourist area have contributed towards this outcome. However, this improvement was not benefitting people as much as it could; people were mostly using private health facilities because they think that medicines from public health facilities are too limited for their health needs and they have doubts about the quality of medicines. Inappropriate handling and use of medicines were found at both the health facility and household levels and this was linked with the pluralistic healthcare system, socio-economic facets of villages and socio-behavioural aspects of the villagers.

Although the government is the main stakeholder in health in the area, this study shows that other stakeholders such as the local community, health aid agencies and tourism contributed significantly to improving access to medicines and health services. Thus, to improve the public perception about quality of medicines and the number of essential medicines and health services, and promote appropriate use of medicines, a joint approach involving all the stakeholders such as government, community, health aid agency and tourism sectors is required.

## Additional file

Additional file 1: **Appendix I.** Access to medicines health facility based study format. **Appendix II.** Semi structured interview format on “Households’ medicines use”. **Appendix III.** Key informants’ interview format on access to medicines and stakeholders of health. (DOCX 35 kb)

## Abbreviations

EHCS: Essential Health Care Services
INGO: International Nongovernment Organization
NGO: Nongovernment Organization
NHSSP: Nepal Health Sector Support Programme
PHC: Primary Health Care
UN: United Nations
VDC: Village Development Committee
WHO: (Staff from an INGO #1) World Health Organization
