# Supplementary material for: Access to and use of medicines in the Annapurna region of Western Nepal and possible impacting factors
Source: J Pharm Policy Pract. 2019 Jun 4;12:11. doi: 10.1186/s40545-019-0172-3 (PMC6547500; doi:10.1186/s40545-019-0172-3)
Supplement: Supplementary file 1 — Appendix I. Access to medicines health facility based study format. Appendix II. Semi structured interview format on “Households’ medicines use”. Appendix III. Key informants’ interview format on access to medicines and stakeholders of health. (DOCX 35 kb) [file 40545_2019_172_MOESM1_ESM.docx]

## Appendix I: Access to medicines health facility based study format

[Availability- based on health facility visit, Affordability, Accessibility and Acceptability-based on exit interview with health service users]

1. Health facility details

| Health facility name: | | Village Development Committee: | |
| --- | --- | --- | --- |
| Population of the VDC: | | Patient/Per Day: | |
| Opening hours (Summer/winter) | | Date: | |
| Qualification/training of prescriber: | | | |
| Qualification/training of dispenser: | | | |
|  | | | |
| Physical condition of health facility | In proper order | | Need maintenance |
| Number of rooms in the health facility: | | | |
| Provision of toilet and tap for washing purpose for the patient : | | | |
| Provision of clean drinking water for the patient: | | | |
| Sitting arrangement for waiting patients: | | | |
| Number of staff in the health facility: | | | |
| Community managed separate drug scheme: | | | |

1. Access to medicines I

| Availability (Based on tracer medicines developed by the MoHP, GoN) | | | | |
| --- | --- | --- | --- | --- |
| % of medicines available | | % of expired medicines present | | |
|  | |  | | |
| Drug information sources(with names): | | | | |
|  | | | | |
|  | | | | |
| S.N. | Tracer medicines  [A] | | In stock, Yes=1, No=0 [B] | Expired medicines  Yes=1, No=0 [C] |
| Core medicines in tracer medicines list | | | | |
| 1 | Albendazole 400mg | |  |  |
| 2 | Aluminium hydroxide+ Magnesium hydroxide 250mg | |  |  |
| 3 | Amoxicillin 250mg | |  |  |
| 4 | Amoxicillin 150mg (Dispersible tab) | |  |  |
| 5 | Chloramphenicol 1% (eye solution) | |  |  |
| 6 | Ciprofloxacin 250mg | |  |  |
| 7 | Sodium lactate (Infusion solution) | |  |  |
| 8 | DMPA 150mg (Family planning) | |  |  |
| 9 | Ferrous salt+ Folic acid 60.04mg | |  |  |
| 10 | Gamma benzene hexachloride 1% | |  |  |
| 11 | Gentamycin 80mg/2ml (injection) | |  |  |
| 12 | Hysocine butylbromide 10mg | |  |  |
| 13 | Metronidazole 400mg | |  |  |
| 14 | Paracetamol 500mg | |  |  |
| 15 | Oral rehydration salt | |  |  |
| 16 | Povidione iodine 5% | |  |  |
| 17 | Sulfamethoxazole+ Trimethoprim (100+20mg) | |  |  |

Access to medicines I *continued*

| S.N. | Tracer medicines  [A] | In stock, Yes=1, No=0 [B] | Expired medicines  Yes=1, No=0 [C] |
| --- | --- | --- | --- |
| 18 | Zinc sulphate 20mg |  |  |
| Supplementary (send as per local target during the national campaign every 6 month) | | | |
| 19 | Vitamin A 200,000 IU | (6 monthly program) |  |
| Supplied during the National Vitamin A Programme only | | | |
|  | DPT, HepB, Hip (Vaccine) |  |  |
| (Not recorded in record book but maintained in a separate activity log book of Dhampus SHP. Supply was regular during the National Immunization Programme) | | | |
|  |  | B^1^= | C^1^= |
|  |  | B^2^= of medicines available  = B^1^/19*100 | C^2^ = % of expired medicines present  = C^1^/19*100 |
| The list of 20 medicines has been identified by the MoHP, Nepal as tracer medicines and is printed in this survey form.  Mark 1 if any quantity of any dosage form of medicine is in stock in the day of facility visit and 0 if the medicine is not in the stock in the day of visit. Calculate the percentage availability of medicines dividing B^1^ by 20and multiply it with 100.  For all medicines in stock check the expiry date of the medicines. If any amount of medicine is expired mark 1 and 0 if not. Calculate the percentage of expired medicines present by dividing C^1^ by 20 and multiply it with 100. | | | |

Access to medicines I *continued*

| Are there any special package for Dalit, women, children and neglected diseases patient? (Special package can be reproductive healthcare package, special medication campaign for children, neonatal care facility for women etc.) | Yes = 1  No= 0 |
| --- | --- |
|  |  |
| Number of essential medicines supplied to health post |  |

1. Access to medicines II

| Availability (Based on tracer medicines developed by the MoHP, GoN) | | | | | |
| --- | --- | --- | --- | --- | --- |
| Medicines with records covering at least 6 months within the past 12 months | | | | | |
| S.N. | Tracer medicines  [A] | Records cover at least 6 months within the past 12 months  Yes=1, No=0 [B] | Only collect data for medicines with records covering at least 6 months within the past 12 months | | |
|  |  |  | Number of days out of stock  [C] | Number of days covered by the review (180 to 365 days) [D] | Eqvt. number of days of stock out per year  [E] = C x 365 ÷ D |
| Core essential medicines (in the tracer medicines list) | | | |  |  |
| 1 | Albendazole 400mg |  |  |  |  |
| 2 | Aluminium hydroxide+ Magnesium hydroxide 250mg |  |  |  |  |
| 3 | Amoxicillin 250mg |  |  |  |  |
| 4 | Amoxicillin 150mg (Dispersible tab) |  |  |  |  |
| 5 | Chloramphenicol 1% (eye solution) |  |  |  |  |

Access to medicines II *continued*

| S.N. | Tracer medicines  [A] | Records cover at least 6 months within the past 12 months  Yes=1, No=0 [B] | Only collect data for medicines with records covering at least 6 months within the past 12 months | | |
| --- | --- | --- | --- | --- | --- |
|  |  |  | Number of days out of stock  [C] | Number of days covered by the review (180 to 365 days) [D] | Eqvt. number of days of stock out per year  [E] = C x 365 ÷ D |
| 6 | Ciprofloxacin 250mg |  |  |  |  |
| 7 | Sodium lactate (Infusion solution) |  |  |  |  |
| 8 | DMPA 150mg (Family planning) |  |  |  |  |
| 9 | Ferrous salt+ Folic acid 60.04mg |  |  |  |  |
| 10 | Gamma benzene hexachloride 1% |  |  |  |  |
| 11 | Gentamycin 80mg/2ml (injection) |  |  |  |  |

Access to medicines II *continued*

| S.N. | Tracer medicines  [A] | Records cover at least 6 months within the past 12 months  Yes=1, No=0 [B] | Only collect data for medicines with records covering at least 6 months within the past 12 months | | |
| --- | --- | --- | --- | --- | --- |
|  |  |  | Number of days out of stock  [C] | Number of days covered by the review (180 to 365 days) [D] | Eqvt. number of days of stock out per year  [E] = C x 365 ÷ D |
| Core essential medicines (in the tracer medicines list) | | | |  |  |
| 12 | Hysocine butylbromide 10mg |  |  |  |  |
| 13 | Metronidazole 400mg |  |  |  |  |
| 14 | Paracetamol 500mg |  |  |  |  |
| 15 | Oral rehydration salt |  |  |  |  |
| 16 | Povidione iodine 5% |  |  |  |  |
| 17 | Sulfamethoxazole+ Trimethoprim (100+20mg) |  |  |  |  |
| 18 | Zinc sulphate 20mg |  |  |  |  |

Access to medicines II *continued*

| S.N. | Tracer medicines  [A] | Records cover at least 6 months within the past 12 months  Yes=1, No=0 [B] | Only collect data for medicines with records covering at least 6 months within the past 12 months | | | |
| --- | --- | --- | --- | --- | --- | --- |
|  |  |  | Number of days out of stock  [C] | Number of days covered by the review (180 to 365 days) [D] | | Eqvt. number of days of stock out per year  [E] = C x 365 ÷ D |
| Supplementary (send as per local target during the national campaign every 6 month) | | | | | | |
| 19 | Vitamin A 200,000 IU |  |  |  |  | |
| Supplied during the National Vitamin A Programme only | | | | | | |
|  | DPT, HepB, Hip (Vaccine) |  |  |  |  | |
| (Not recorded in record book but maintained in a separate activity log book of Dhampus SHP. Supply was regular during the National Immunization Programme) | | | | | | |
|  |  | [B1] = Sum of B = | C^1^= 0 |  | [E1] = Sum of E = 0 | |
|  |  | [B2] =  % of adequate records | C^2^ = % of expired medicines present |  |  | |

Access to medicines II *continued*

| [F] = Average number of stock-out days = E1 ÷ B1 = 0/19 = 0 |
| --- |
| [B] Go through the stock cards and indicate which medicines have records covering at least 6 months within the previous 12 months. Add the total at the bottom [B1]. Calculate the percentage of medicines with adequate records [B2] by dividing the number of medicines with records covering at least 6 months [B1] by X and multiplying by 100. |
| [C] The review should cover 6-12 months. Go through the stock cards covering the review period. Indicate the number of days each medicine was not available or marked “0” on the card. A medicine is considered in stock if any quantity of it is available in generic or branded form. |
| [D] Indicate the number of days actually reviewed for each medicine. |
| [E] Compute the equivalent number of stock-out days per year for each medicine by multiplying the number of days out of stock [C] by 365 and dividing by the number of days covered by the review [D]. Write the total number of stock-out days [E1]. |
| [F] Calculate the average number of stock-out days by dividing the total number of stock-out days [E1] by the total number of medicines reviewed [B1]. |

1. Adequate conservation conditions and handling of medicines in storeroom and dispensing area

| Facility: | | Date: | |
| --- | --- | --- | --- |
| Checklist | Store room [A]  True=1, False=0 | | Dispensing Area / Room [B]  True=1, False=0 |
| 1. Provisions of false ceiling (control temperature), windows (with curtain) and air vents. |  | |  |
| 1. Provisions to control medicines from moisture and direct sunlight. |  | |  |
| 1. Medicines are not stored directly on the floor. |  | |  |
| 1. Enough selves and racks to store medicines |  | |  |
| 1. Medicines are stored in a systematic way (e.g. alphabetical, pharmacological). |  | |  |
| 1. Medicines are stored first-expiry-first out (FEFO). |  | |  |
| 1. There is no evidence of pests in the area. |  | |  |
| 1. Provision of refrigerator. |  | |  |
|  | [A1] =Sum of A | | [B1] =Sum of B |
|  | [A2] = Score =  A1 ÷ 8 x 100 = | | [B2] = Score =  B1 ÷ 8 x 100 = |

Adequate conservation conditions and handling of medicines in storeroom and dispensing area *continued*

***Notes:***

[A] Indicate “1” if all parts of the statement are true for the storeroom and “0” if any part of it is false. Sum the total number of true statements in [A1]. Calculate the score for the storeroom [A2] by dividing the sum of true statements [A1] by 8 and multiplying by 100.

[B] Indicate “1” if all parts of the statement are true for the dispensing room and “0” if any part of it is false. Sum the total number of true statements in [B1]. Calculate the score for the dispensing room [B2] by dividing the sum of true statements [B1] by 8 and multiplying by 100.

* It may be necessary to look elsewhere in the facility for some of the criteria (e.g. refrigerator).

1. Health post incharge’s and staffs’ remarks about

| Supply of medicines at SHP |
| --- |
| Accessibility (Issue of Dalit’s access) |
| Dispensing of medicines |
| People’s participation/contribution in public health programs |
| Village development committee’s and local people’s contribution to the HP |
| Involvement/support from health post management committee |
| Support from INGOs/NGOs/aid |
| District health office’s support/contribution |
| Community drug program |
| Other comments |

1. Exit interview with health services users

| Accessibility | | |
| --- | --- | --- |
| Health facility | Health service users: | |
| Date: | Dalit/non-Dalit: | |
| How long did it take for the patient to reach the health facility today? [D]  D^1^= sum of 1=  D^2^= sum of 2 =  D^3^= sum of 3 = | | 1. < 30 min |
|  |  | 2. 31-60 min |
|  |  | 3. >60 min |
| Is there any alternative health facility in your village? (Alternative facility can be village health clinic, private pharmacy etc.) E^1^ = Sum of E | | [E] Yes = 1  No= 0 |
| How long does it take for you to get your treatment in health post? (starting from the waiting time to diagnosis till you get the medicines and leave the health facility)  F^1^=sum of 1=  F^2^= Sum of 2= | | [F] 1. <30 min  2. 31-60 min |
| For Dalit’s only | | |
| Do you have to face any kind of discrimination in accessing health facility due to your socio-cultural background?  G^1^= sum of 1=  G^2^= sum of 2= | | [G] 1. Yes =  2. No=0 |

Exit interviews with health services user *continued*

| Affordability | |
| --- | --- |
| Do you have to pay user fee? | Yes =1, No= 0 |
| How many days of work the patient miss due to illness? (Calculate each day’s wage based on local wage rate per day, multiply by the number of days missed and convert it to USD/per day) | [H]= 1 |
| [H1] = sum of I  Average= I1/total no of response = (average amount, lost due to illness) | |
| Acceptability | |
| Are you satisfied with this health post visit (latest visit)? | Yes=1, No=0 |
| Are you satisfied with the quality of medicines provided by the health post? | Yes=1, No=0 |
| Does the patient have any comments about the free drug scheme? | |

1. Quality of product and services

| Collection of information on medicines prescribed and dispensed | | |
| --- | --- | --- |
| Patient # | Sex: M/F | |
| Age: <5 5-15 16-60 >60 | | |
| Number of medicines in the prescription | |  |
| Medicines adequately labelled | | Yes= 1, No=0 |
| Patient knows how to take medicines | | Yes= 1, No=0 |
| Does the prescription include antibiotics? | | Yes= 1, No=0 |
| Medicines | |  |
|  | |  |
|  | |  |
|  | |  |
|  | |  |
|  | |  |

## Appendix II: Semi structured interview format on “Households’ medicines use”

| Responder number: | Interviewer: | | |  |
| --- | --- | --- | --- | --- |
| Village: | Date of interview: | | |  |
| Socio-demographic information | | | |  |
| Family size: | Primary source of income: | | |  |
| Education level of the head of the family: | Number of children: | <5 years | 6-16 years |  |
| 1. What are the different types of medicines you use in your house? (Systems of medicine) | | | | |
| 1. Where do you get the medicines if someone in your family falls ill? (only allopathic medicines) | | | | |
| 1. Where do you often go for treatment? | | | | |
| 1. Is any member of your family currently taking traditional medicine? If yes for what diseases? | | | | |

Households’ medicines use *continued*

| 1. How do you take traditional medicines?   Duration (How long?):  Dose (What quantity?):  Method of administration:  Concomitant use (Is the traditional medicines used together with other medicines?): | |
| --- | --- |
| Information on medicines available in the households | |
| 1. Do you have any medicine/s currently used by any member of your family?   (If yes, request them to show all the medicine and fill in the following information) | |
| 1. Name of medicine: | For whom: |
| Used for: | Labelling: |
| Expiry date: | Storage: |
| Source: |  |
| 1. Name of medicine: | For whom: |
| Used for: | Labelling: |
| Expiry date: | Storage: |
| Source: |  |

Households’ medicines use *continued*

| 1. Do you have any medicines in the households kept specifically for future use? (If yes, ask them to show all the medicines and fill in the following information) | |
| --- | --- |
| 1. Name of medicine: | Labelling: |
| Do you know the use of this medicine? | Expiry date: |
| Is it an antibiotic: | Storage: |
| Physical condition of the medicine: |  |
| 1. Name of medicine: | Labelling: |
| Do you know the use of this medicine? | Expiry date: |
| Is it an antibiotic: | Storage: |
| Physical condition of the medicine: |  |
| 1. Do you have any **medicine/s left over** in your house? (If yes, request them to show all medicines and fill in the following information) | |
| 1. Name of medicine: | Expiry date: |
| Is it an antibiotic: | Disposal: |
| Is it kept for future purpose? |  |
| 1. Name of medicine: | Expiry date: |
| Is it an antibiotic: | Disposal: |
| Is it kept for future purpose? |  |

Households’ medicines use *continued*

| Information on medicines use pattern |
| --- |
| 1. a. Do you or the sick persons go to the health post if they get sick? |
| b. If not, do you send someone on your behalf to get medicines for you? (If yes, When? And Why?) |
| 1. Do you or the sick persons take the medicine in the same manner as told by the doctor/health care provider?   Frequency:  Dose:  Duration:  (If not, why don’t you follow the medicine schedule?) |
| 1. Do pharmacists/dispensers tell you about the expiry date of the medicines? |
| 1. Do you check the expiry date of medicine before taking it? (Yes/No/How often) |
| Information on chronic diseases |
| 1. a. Do any of the members of your family have any chronic diseases (diseases like hypertension, diabetes, asthma etc. as told by the doctor? |
| If yes, can you name the disease? |

Households’ medicines use *continued*

| 1. If yes is he/she taking the medicines? |
| --- |
| 1. If not, why is he/she not taking the medicines? |
| 1. If yes, how are you financing the cost of treatment of the person? |
| 1. If yes, on an average, how much money do you spend monthly in buying his/her medicine? |
| Information on the accessibility of nearest healthcare facility |
| 1. How much time does it take from your house to reach the health post? |
| 1. How much time does it take from your house to reach the nearest hospital? |
| 1. How do you take seriously ill person to hospital? |
| 1. What is the cost of transportation to the nearest hospital? |
| For Dalits only |
| 1. Do you have any problems in accessing the health post service? (If yes please explain) |
| 1. How do the healthcare providers treat you as a client of the health post? |
| 1. Any comments on the state of access to medicines in your village? |

## Appendix III: Key informants’ interview format on access to medicines and stakeholders of health

Key informants interview representing

- Government (health sector)
- Local community
- Health aid agency
- Tourist specific health facility
- Other stakeholders

1. Government (health sector)

| Interviewee: | **Government** |
| --- | --- |
| Post: | District (Public) Health Officer (D(P)HO) |
| Date: | Logistics Officer |
| Place: | Health Post Incharge |
|  | Public health authority/officers |
| 1. Is people’s access to medicines one of your (District health office/Health Post) program’s priorities? How is that reflected in the DHO/Health Post functioning? | |
| 1. What special program has the government brought to improve people’s access to medicines and health services in the Annapurna region? | |
| 1. How do you see the state of access to medicines in the Annapurna region? | |
| 1. What is your opinion about logistics, distribution and supply of essential medicines in the Annapurna region? | |
| 1. What is your experience with the community drug program? | |
| 1. What is your experience with the current free drug scheme? | |
| 1. How do you see the free drug scheme in comparison to the community drug program? | |
| 1. What is the contribution of health post management committee in health post activities? | |

Government (health sector) *continued*

| 1. Your comments on improving access to medicines in rural areas of Nepal. |
| --- |
| 1. Government as main stakeholder i.e. provider of health services and medicines and questions/issues regarding rural health services and medicines delivery. |

1. Local community

| Interviewee: | Community |
| --- | --- |
| Occupation: | Health Post Management Committee |
| Date: | Village Development Committee |
| Place: | Female Community Health Volunteers |
|  | Other community members |
| 1. How do you see local people’s involvement in health care activities? | |
| 1. What was your experience of managing the community drug program in your VDC? | |
| 1. How has the village development committee helped in improving access to health services and medicines in the community? | |
| 1. How do you see the contribution of local community in public health programs like Vitamin A program, immunisation campaign, albendazole medication campaign etc.? | |
| 1. Have you ever faced any kind of problem in implementing public health programmes in this village due to the sociocultural background of the villagers? | |
| 1. Do you take part in any of the essential medicines related activities of health post? | |
| 1. How is the local revenue generated from tourism in the Annapurna region used? | |

Local community *continued*

| 1. What is your view on the access to medicines after the initiation of free drug scheme? |
| --- |
| 1. Do the tourists provide any kind of direct or indirect health/medical support to the villages of the Annapurna region? |
| 1. Your comments on the state access to medicines and ways to improve it in the Annapurna region? |
| 1. What are the activities you do as a female community health volunteer?   (for FVHCs only) |

1. Health aid agencies (INGO/NGOs, donors, trusts etc.)

| Interviewee: | Date: |
| --- | --- |
| Post: | Place: |
| Health Aid Agency’s name: | |
| 1. General questions | |
| 1. What is the basic working area and function of your organization? | |
| 1. How has your organisation contributed towards the healthcare sector, especially in this western region? | |
| 1. What is your experience and perception of the involvement of your organisation in the healthcare activities in the western region? | |
| 1. Has your organization ever donated medicines or health equipment to health facilities of the western region? | |
| 1. What has been your experience with the community drug program? | |
| 1. In your opinion, was the community drug program successful in fulfilling the medicines need of the local people? | |
| 1. Your comments on the state of access to medicines and health services in the western region of Nepal. | |

Health aid agencies *continued*

| 1. Organisation specific questions |
| --- |
| 1. Do you think your organisation’s programmes are in sync with the government program and policies (particularly the MoHP’s policy and programmes)? |
| 1. How does your organisation perform procurement and management of medicines? (any difficulties? especially, when your organisation is the only tertiary care facility for neglected tropical diseases in Nepal) |
| 1. Sustainability of the healthcare projects launched by your organisation? (In view of the treatment of neglected tropical diseases, ambitious spinal cord injury and rehabilitations programme, multidrug resistant TB and other sophisticated healthcare programmes) |
| 1. Any prospects of collaboration with the government? (such as carrying out any projects together with government or handing over any project to them) |
| 1. In view of your organisations’ long experiences in public health sector in Nepal, how do you see the socioeconomic changes and its linkage with healthcare development? |
| 1. Any problems/issues your organisation had to face while working with the community? |

1. Tourism/tourist specific health facility

| 1. Please tell me about the general profile, objective and scope (kinds of health services) of this medical aid post/health facility? |
| --- |
| 1. How has this medical aid post/health facility contributed towards the medicine need of the local people? |
| 1. What is the contribution of this medical aid post/health facility to local people’s healthcare? |
| 1. Do the tourists contribute (donate) to local healthcare? (If yes, probe to know how and by what means?) |
| 1. How has the people’s beliefs/perception regarding medicines changed after your health facility/organisation started serving in this region? |
| 1. How do you carry out healthcare operation and manage the financial aspects? |
| 1. How do you manage medicines supply for your health facility in the Annapurna region? |
| 1. Your comments on the state of access to medicines in the Annapurna region and the prospects of new projects in health sector in this region? |
| 1. What are the commonly occurring diseases in this high altitude areas of the Annapurna region? |
| 1. General comments about improving access to healthcare and medicines. |
